# Efficacy and Safety of AbobotulinumtoxinA for the Treatment of Glabellar Lines in Chinese Patients: A Pivotal, Phase 3, Randomized, Double-Blind and Open-Label Phase Study

**DOI:** 10.1007/s00266-022-03164-3

**Published:** 2022-12-19

**Authors:** Yan Wu, Fang Fang, Wei Lai, Chengxin Li, Li Li, Quanzhong Liu, Jianyun Lu, Xiaowen Pang, Jiaming Sun, Xiaofeng Shi, Philippe Picaut, Inna Prygova, Bill Andriopoulos, Qiuning Sun

**Affiliations:** 1 Peking University First Hospital, No. 8 Xishiku Street, Xicheng, Beijing, 100034 China; 2 Institute of Dermatology, Chinese Academy of Medical Sciences, Nanjing, China; 3 Department of Dermatology, The Third Affiliated Hospital of Sun Yat-Sen University, Guangzhou, China; 4 General Hospital of People’s Liberation Army (301 Hospital), Beijing, China; 5 West China Hospital, Sichuan University, Chengdu, China; 6 Tianjin Medical University General Hospital, Tianjin, China; 7 Third Xiangya Hospital of Central South University, Changsha, China; 8 PLA Air Force General Hospital, Beijing, China; 9 Union Hospital, Tongji Medical College, Huazhong University of Science and Technology, Wuhan, China; 10 Ipsen, Shanghai, China; 11 Ipsen, Cambridge, MA USA; 12 Ipsen, Boulogne-Billancourt, France; 13 Galderma, Uppsala, Sweden; 14 Peking Union Medical College Hospital, Beijing, China

## Abstract

**Background:**

Various botulinumtoxinA formulations are approved for glabellar lines treatment worldwide, including abobotulinumtoxinA (Dysport^®^).

**Objectives:**

Assess abobotulinumtoxinA superiority versus placebo and non-inferiority versus active comparator (onabotulinumtoxinA; Botox^®^), for the treatment of Chinese patients with moderate/severe glabellar lines.

**Methods:**

Phase 3, randomized study (NCT02450526) comprising a double-blind (cycle 1) phase and an open-label (cycles 2−5) phase. Patients received abobotulinumtoxinA 50 units or matching placebo (5:1), active comparator (onabotulinumtoxinA 20 units) or matching placebo (5:1). In cycles 2–5, eligible patients were retreated with abobotulinumtoxinA only. Responders had glabellar lines of none/mild severity. Primary endpoint: responder rates at cycle 1, day 29 at maximum frown with abobotulinumtoxinA versus placebo (for superiority; by investigator’s live assessment [ILA] and subject’s self-assessment [SSA]), and versus active comparator (for non-inferiority; by ILA). Treatment-emergent adverse events were recorded.

**Results:**

Overall, 520 patients were randomized. Superiority and non-inferiority, respectively, were demonstrated for abobotulinumtoxinA versus placebo (ILA, SSA; both *p *< 0.0001) and abobotulinumtoxinA versus active comparator. AbobotulinumtoxinA efficacy was maintained over open-label cycles; median time to onset of efficacy was 2.0 days. After 6 months, 17% of patients treated with abobotulinumtoxinA remained responders. AbobotulinumtoxinA was well-tolerated. Safety results were in line with the known profile of abobotulinumtoxinA; adverse events rate decreased with repeated treatment.

**Conclusions:**

After a single injection, abobotulinumtoxinA demonstrated superiority versus placebo and non-inferiority versus onabotulinumtoxinA for the treatment of moderate-to-severe glabellar lines in Chinese patients. Multiple injections of abobotulinumtoxinA demonstrated efficacy and safety in the treatment of glabellar lines in Chinese patients.

**Level of Evidence I:**

This journal requires that authors assign a level of evidence to each article. For a full description of these Evidence-Based Medicine ratings, please refer to the Table of Contents or the online Instructions to Authors www.springer.com/00266.

## Plain Language Summary

Botulinum toxin injections can be used to smooth frown lines that appear between the eyebrows (known as glabellar lines) in patients who have moderate or severe frown lines. This study looked at how injections of a botulinum toxin (abobotulinumtoxinA [aboBoNT-A]) could help with smoothing frown lines in patients from China compared with an injection of another botulinum toxin called onabotulinumtoxinA (onaBoNT-A) or placebo (saltwater, no treatment). The study included 520 patients from China, 18–65 years old, who had moderate or severe frown lines. All patients received a first injection of either aboBoNT-A, onaBoNT-A, or saltwater, and were studied for 12 weeks. After the first injection, patients could receive up to four more injections of aboBoNT-A, given at 12-week intervals, if their frown lines became moderate or severe again. Most patients (92%) had not previously received any botulinum toxin injections. The results showed that single and repeat injections of aboBoNT-A helped to smooth moderate and severe frown lines. The researchers found that after a single injection, aboBoNT-A was superior to no treatment and was similar to onaBoNT-A. Patients recorded a response to aboBoNT-A after 2 days and the response lasted for 6 months in 17% of patients. The effect on frown lines was maintained after repeat injections and aboBoNT-A was well tolerated by patients. These results suggest that aboBoNT-A is a suitable treatment for smoothing frown lines in patients from China with moderate to severe frown lines.

## Introduction

A number of botulinum toxin type A (BoNT-A) formulations have been approved for the treatment of glabellar lines (wrinkles that appear between the eyebrows, also known as frown lines) worldwide, with additional indications, such as lateral canthal lines (also known as crow’s feet), approved in some countries [1, 2]. BoNT-A reduces glabellar lines by suppressing the muscular activity of the glabellar area to temporarily relax the procerus and corrugator muscle complex [3–9].

AbobotulinumtoxinA (aboBoNT-A; Dysport^®^, Ipsen Ltd.) is approved at a recommended dose of 50 units (U) (approved in China since June 2020) for the treatment of glabellar lines in over 80 countries worldwide (including in Europe and the USA) [10, 11]. Many studies have demonstrated that aboBoNT-A has a good safety profile, and a predictable and high rate of efficacy when treating glabellar lines, including a fast onset of action, prolonged duration of action, and high rate of patient satisfaction [7, 12–19]. However, limited clinical data are available on the safety and efficacy of aboBoNT-A for the treatment of moderate-to-severe glabellar lines in the Chinese population.

To address this gap, the aim of the current study was to demonstrate the short- and long-term efficacy and safety of repeated administrations of aboBoNT-A 50 U in the treatment of moderate-to-severe glabellar lines in Chinese patients. The superiority of aboBoNT-A compared with placebo, and non-inferiority of aboBoNT-A compared with an active comparator (onabotulinumtoxinA [onaBoNT-A]; Botox^®^, Allergan Inc.), was assessed based on the requirements of the China Food and Drug Administration.

## Materials and Methods

### Study Design and Intervention

This was a phase 3, multicenter, randomized study comprising a double-blind phase followed by an open-label phase (ClinicalTrials.gov, NCT02450526), conducted at ten centers in China between April 2015 and September 2017. Since the volumes to be administered per injection site differed for aboBoNT-A and the active comparator onaBoNT-A, two separate matching placebos (i.e., one each for aboBoNT-A and onaBoNT-A) were used to maintain the blinding status at the product level. Hence, patients were randomized into four treatment groups to receive 1 cycle of aboBoNT-A (Group A), matching aboBoNT-A placebo (Group B), active comparator (onaBoNT-A; Group C), or matching comparator placebo (Group D). Patients were randomized to receive aboBoNT-A or comparator in a 3:1 ratio. Within the aboBoNT-A group, patients received either the treatment or placebo in a 5:1 ratio (Group A: Group B). Patients within the comparator group also received active treatment or placebo in a 5:1 ratio (Group C: Group D). Randomization was stratified by sex and baseline severity of glabellar lines at maximum frown, measured by investigator’s live assessment (ILA), to minimize bias in treatment allocation within the specified levels of these factors, thus avoiding confounding the treatment effect. Randomization was performed by an independent statistician in blocks based on computer-generated randomization lists and was managed using an interactive web response system.

Study treatments were administered in five predefined sites across the glabellar area: two injections into both corrugator muscles and one into the procerus muscle near the nasofrontal angle. Patients received aboBoNT-A 50 U (10 U [0.05 mL] per injection site), onaBoNT-A 20 U (4 U [0.1 mL] per injection site), matching aboBoNT-A placebo (containing the excipients from aboBoNT-A reconstituted with 1.5 mL of sodium chloride solution), or matching onaBoNT-A placebo (1.5 mL of sodium chloride solution). After treatment, all patients attended follow-up visits on days 8, 29, 57, and 85. After the double-blind treatment cycle (cycle 1), patients received a maximum of four additional cycles (cycles 2−5) with open-label aboBoNT-A 50 U at intervals of  ≥ 84 days (12 weeks) between each cycle, depending on individual patient duration of response to treatment. Patients could start the next treatment cycle (retreatment) if their glabellar line severity had returned to moderate or severe, regardless of the severity of their glabellar lines at baseline. Patients who were ineligible for retreatment were evaluated every 28 days at additional follow-up visits in each treatment cycle until they were eligible for retreatment. Patients completed the study when they had attended the day 85 visit after their fifth injection (including cycle 1), or when they had been in the whole study for 15 months.

### Patient Population

Inclusion criteria were Chinese patients between 18 and 65 years of age, with moderate (Grade 2) or severe (Grade 3) wrinkles of vertical glabellar lines at maximum frown at baseline per the ILA, a validated four-point photographic scale to assess the severity of glabellar lines (where 0 = no wrinkles and 3 = severe wrinkles), and the subject’s self-assessment scale (SSA; a four-point categorical scale to assess the severity of glabellar lines, where 0 = no wrinkles and 3 = severe wrinkles). All patients were BoNT-A-naïve or had received their most recent BoNT-A treatment in any muscle of the face  > 1 year before screening. Exclusion criteria included any previous or planned treatments or surgeries that may have interfered with the evaluation of the study results and an inability to substantially lessen the glabellar lines by physically spreading them apart or a lack of capacity to frown. In addition, patients were ineligible if they had any current conditions (including facial conditions), were using any concomitant medications that could interfere with the safety, conduct, or outcomes of the study, or had a known allergy or hypersensitivity to BoNT-A or excipients. Female patients who were pregnant, lactating, or of childbearing potential but not willing to use contraception were also unable to participate.

### Endpoints and Assessments

The primary efficacy endpoints were the proportion of responders treated with aboBoNT-A at cycle 1, day 29 at maximum frown, as measured by the ILA and SSA to test for superiority compared with placebo, and measured by the ILA to test for non-inferiority compared to active comparator. A responder was defined as having a severity grade of none (0) or mild (1) at maximum frown.

The secondary efficacy endpoints included the proportion of responders treated with aboBoNT-A compared with placebo, as evaluated by an independent reviewer’s assessment (IRA) of photographs of the patient’s glabellar lines at maximum frown at cycle 1, day 29 (using a four-point photographic scale, where 0 = none and 3 = severe). The mean subject’s global assessment (SGA) score (nine-point scale from  + 4 [100% improvement] to 0 [no change] to −4 [100% worsening]) at cycle 1, day 29 and the proportion of responders at cycle 1, day 29 with respect to the SGA score (a responder based on the SGA scale was defined as having a grade of at least  + 2 [50% improvement]) were also assessed.

Further endpoints in cycle 1 were the proportion of responders measured by the ILA and SSA at maximum frown and by the ILA at rest, and the proportion of responders measured by the SGA score, measured at all study visits except day 29. Additional assessments were the proportion of responders according to the IRA of photographs at day 85; the correlation between the ILA and SSA of glabellar lines at maximum frown at baseline and day 29; the mean change from baseline in the patient’s self-perception of age (categories: ‘*I look like my current age’*; ‘*I look _ years younger*’; ‘*I look _ years older*’) at all study visits; and time to onset of treatment response based on the patient’s diary card. (Patients were asked to record their assessment of the study treatment response from days 2 to 8.) From cycle 2 onwards, the proportion of responders measured by the ILA and SSA at maximum frown and the ILA at rest, and with respect to the SGA score, was assessed at all study visits. In addition, the mean change from cycle baseline (day 1 assessment of each treatment cycle) in the patient’s self-perception of age at day 29 of each treatment cycle was assessed.

Safety endpoints included treatment-emergent adverse events (TEAEs), vital signs, clinical laboratory evaluations, and the presence of BoNT-A antibodies.

Results from both the double-blind and open-label phases are presented for patients who received aboBoNT-A, and results from patients who received the active or placebo comparator are limited to baseline, primary endpoint, and safety data.

### Statistical Methods

A sample size of approximately 480 patients was considered appropriate to demonstrate the superiority of aboBoNT-A compared with placebo, the non-inferiority of aboBoNT-A compared to active comparator, and the assessment of aboBoNT-A safety according to the China Food and Drug Administration guidelines. Statistical assumptions were based on previously published literature [20–22].

Efficacy analyses were performed on the modified intent-to-treat population (all randomized patients who received study treatment in  ≥ 1 injection site and had both cycle 1, day 1 [baseline] and cycle 1, day 29 assessments) and on the open-label population (all randomized patients who received any dose of open-label aboBoNT-A; cycles 2–5). The superiority of aboBoNT-A to matching placebo was tested using a multivariate logistic regression model including treatment group, stratification factors of gender and baseline severity score (glabellar lines at maximum frown measured by the ILA), treatment center as an explanatory variable, and responder (yes or no) as a response variable. A two-step hierarchical testing procedure was applied to control the family-wise type I error rate (using 0.025 significance levels). If superiority was demonstrated, non-inferiority was tested one-sided at the significance level of 0.025. If the lower limit of the 95% confidence intervals (CIs) of the treatment difference was  ≥ −15%, aboBoNT-A was considered to be non-inferior compared to the active comparator.

The multivariate logistic regression model was used for analysis of the proportion of responders based on photographs of patient glabellar lines at maximum frown and proportion of responders with respect to SGA score. A linear mixed model (which included stratification factors of gender and baseline severity score, and center as fixed effect) was used to analyze mean SGA scores at cycle 1, day 29; the test was two-sided at the significance level of 0.05. Time to onset of treatment response was analyzed using the Cox model, and mean change from baseline in patient’s self-perception of age was analyzed by mixed model with repeated measures. The agreement between different measures was determined using Spearman’s rank correlation coefficient, weighted kappa statistics, and 95% CIs. Safety endpoints were presented using descriptive statistics.

## Results

### Patient Disposition and Baseline Characteristics

**Fig. 1:**
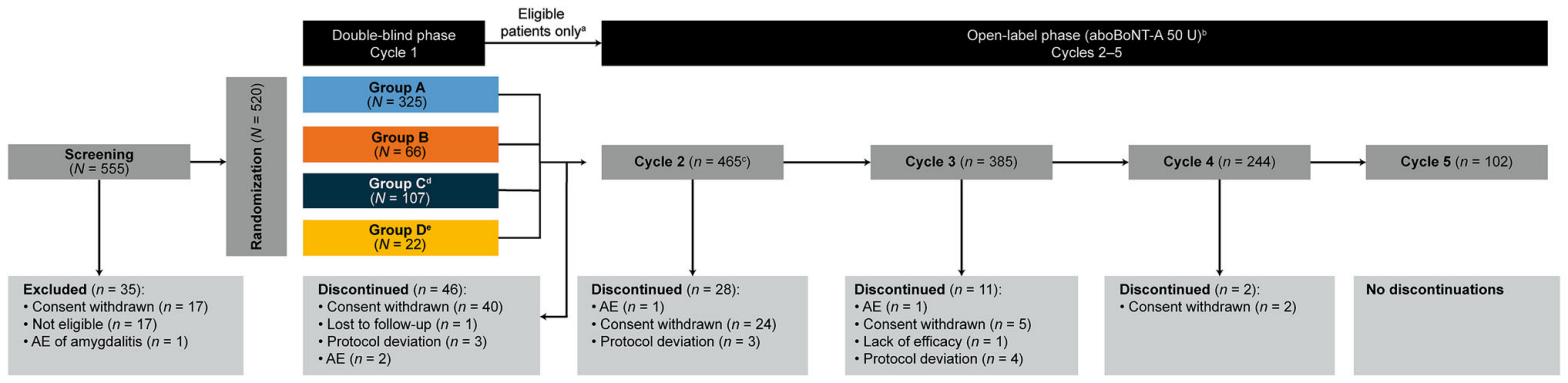
Patient disposition ^a^Patients could be retreated if their glabellar line severity had returned to moderate or severe regardless of the severity at baseline. Patients who were ineligible for retreatment were evaluated every 28 days at additional follow-up visits until they were eligible for retreatment, for 12 months. ^b^Patients progressed to subsequent treatment cycles depending on aesthetic. ^c^Of the 465 patients who entered into cycle 2, 290, 94, 61, and 20 had received aboBoNT-A 50 U, onaBoNT-A 20 U, matching aboBoNT-A placebo, or matching onaBoNT-A placebo, respectively, in cycle 1. One patient was randomized to the matching onaBoNT-A placebo group (Group D) but received treatment of aboBoNT-A 50 U in cycle 1; this patient was counted in the aboBoNT-A 50 U group in the safety population. ^d^OnaBoNT-A 20 U. ^e^Matching onaBoNT-A placebo. AboBoNT-A = abobotulinumtoxinA; AE = adverse event; Group A = patients who received aboBoNT-A 50 U; Group B = patients who received matching aboBoNT-A placebo; Group C = patients who received onaBoNT-A 20 U; Group D = patients who received matching onaBoNT-A placebo; *N* = total number of patients; *n* = number of patients; onaBoNT-A = onabotulinumtoxinA; U = unit

Overall, 555 patients were screened and 520 patients were randomized to receive aboBoNT-A (Group A, *n* = 325), matching aboBoNT-A placebo (Group B, *n* = 66), active comparator (Group C, *n* = 107), or matching comparator placebo (Group D, *n* = 22) (Fig. 1). In total, 432 patients (83.1%) completed the study, with similar discontinuation rates among the treatment groups; the most common reason for discontinuation was withdrawal of consent (13.7%).

Baseline characteristics and demographics are given in Table 1; 86.7% of patients were female; more patients had ‘severe’ than ‘moderate’ appearance of glabellar lines at maximum frown (56.9% compared with 43.1%, respectively), and 92.2% of patients were naïve to previous BoNT-A treatment. The mean (standard deviation [SD]) study duration was 448.0 (106.1) days (range: 11–629 days), and the median duration of exposure (median time to retreatment) during the double-blind period (calculated as the time interval between injection in cycle 1 and the first injection received in the open-label period) was 140.0 days for patients treated with aboBoNT-A in Group A compared with 88.0 days for the placebo Group B.

**Table 1:** Demographic and baseline characteristics

Characteristic	Treatment group^a^				
	Group A	Group B	Group C	Group D	Total
	*N* = 323	*N* = 65	*N* = 106	*N* = 21	*N* = 515
Age, years					
Mean (SD)	45.6 (9.3)	44.2 (9.5)	44.8 (8.2)	42.5 (10.0)	45.2 (9.2)
Range	20–64	24–60	26–63	23–57	20–64
Sex, n (%)					
Male	43 (13.3)	9 (13.8)	13 (12.3)	3 (14.3)	68 (13.2)
Female	280 (86.7)	56 (86.2)	93 (87.7)	18 (85.7)	447 (86.8)
Race, *n* (%)					
Asian	323 (100)	65 (100)	106 (100)	21 (100)	515 (100)
Appearance of glabellar lines at maximum frown, measured by ILA, *n* (%)					
Moderate (Grade 2)	139 (43.0)	29 (44.6)	46 (43.4)	8 (38.1)	222 (43.1)
Severe (Grade 3)	184 (57.0)	36 (55.4)	60 (56.6)	13 (61.9)	293 (56.9)
Botulinum toxin status, *n* (%)					
Naïve	295 (91.3)	61 (93.8)	100 (94.3)	19 (90.5)	475 (92.2)
Non-naïve^b^	28 (8.7)	4 (6.2)	6 (5.7)	2 (9.5)	40 (7.8)

Data are shown from the mITT population. ^a^Treatment group in cycle 1. ^b^Patients who were non-naïve to botulinum toxin had received their most recent BoNT-A treatment more than 1 year prior to screening, in any muscle of the face. Group A = patients who received aboBoNT-A 50 U; Group B = patients who received matching aboBoNT-A placebo; Group C = patients who received onaBoNT-A 20 U; Group D = patients who received matching onaBoNT-A placebo; AboBoNT-A = abobotulinumtoxinA; ILA = investigator’s live assessment; mITT = modified intent-to-treat; *N* = total number of patients; *n* = number of patients with an assessment; onaBoNT-A = onabotulinumtoxinA; SD = standard deviation; U = unit

### Double-Blind Efficacy Results

**Fig. 2:**
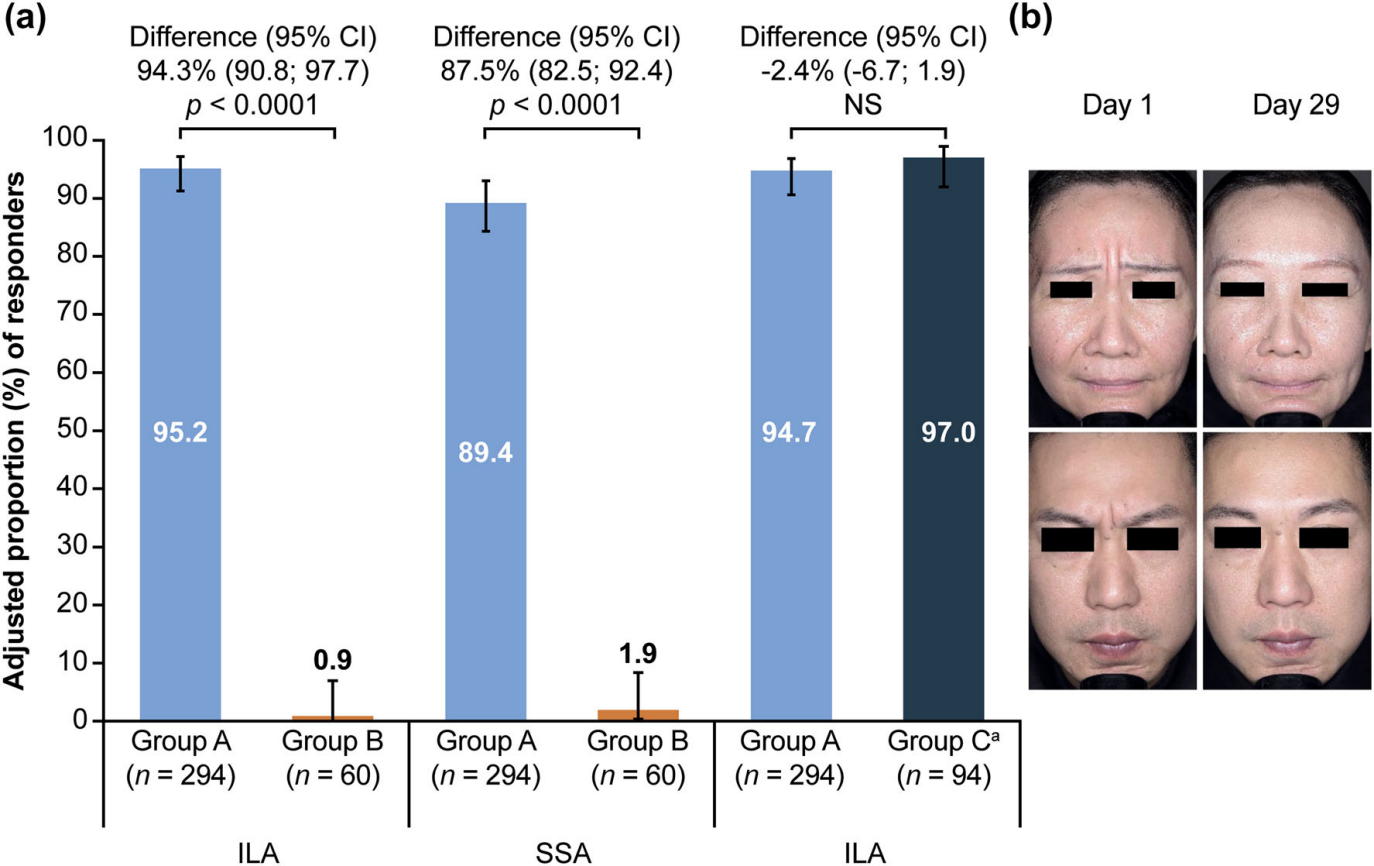
**a** Superiority and non-inferiority analysis—proportion of responders at cycle 1 day 29 of glabellar lines at maximum frown, **b** examples of 2 responders at maximum frown with ‘severe’ glabellar lines at day 1 and ‘none’ on day 29 following aboBoNT-A treatment in cycle 1. Data are shown from the mITT population. ^a^Active comparator. AboBoNT-A = abobotulinumtoxinA; CI = confidence interval; Group A = patients who received aboBoNT-A 50 U; Group B = patients who received matching aboBoNT-A placebo; Group C = patients who received onaBoNT-A 20 U; ILA = investigator’s live assessment; mITT = modified intent-to-treat; NS = not significant; onaBoNT-A = onabotulinumtoxinA; SSA = subject’s self-assessment; U = unit

At cycle 1, day 29 (primary endpoint), 95.2% of patients treated with aboBoNT-A in Group A achieved a response compared with 0.9% of those receiving placebo in Group B (according to ILA: treatment difference 94.3% [95% CI 90.8; 97.7], *p *< 0.0001; according to SSA: treatment difference 87.5% [95% CI 82.5; 92.4], *p *< 0.0001) (Fig. 2), demonstrating the superiority of aboBoNT-A over placebo.

Non-inferiority was demonstrated for aboBoNT-A compared to the active comparator (primary endpoint) with 94.7% compared to 97.0% adjusted proportion of responders in Groups A and C, respectively, at maximum frown at day 29 (according to ILA: treatment difference −2.4 [95% CI −6.7; 1.9]) (Fig. 2A).

**Fig. 3:**
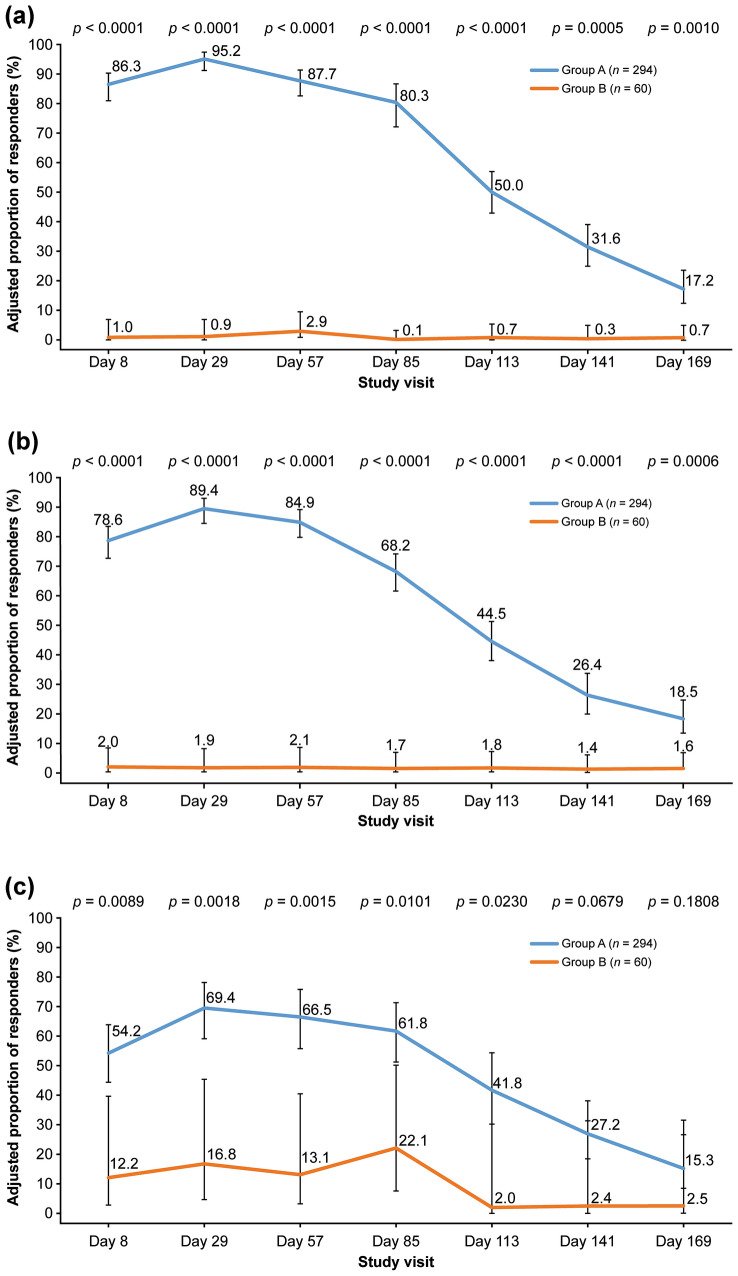
Superiority analysis—proportion of responders by **a** ILA of glabellar lines at maximum frown, **b** SSA of glabellar lines at maximum frown, and **c** ILA of glabellar lines at rest. Data are shown from the mITT population. AboBoNT-A = abobotulinumtoxinA; Group A = patients who received aboBoNT-A 50 U; Group B = patients who received matching aboBoNT-A placebo; ILA = investigator’s live assessment; mITT = modified intent-to-treat; *n* = number of patients; SSA = subject’s self-assessment; U = unit

At all visits during the double-blind period, the adjusted proportion of responders at maximum frown by both the ILA and SSA was statistically significantly greater in the aboBoNT-A group (Group A) compared with placebo (Group B) (all *p *< 0.001) (Fig. 3a and b). The proportion of responders at maximum frown was significantly greater in the active comparator group (Group C) compared with placebo (Group D) at day 29, as assessed by ILA and SSA (*p *< 0.0001).

At maximum frown, 72 (17.2%) of the patients in Group A treated with aboBoNT-A were still responders on day 169 (approximately 6 months since the start of treatment), compared with one patient in the placebo Group B (according to ILA: treatment difference 16.5% [95% CI 11.7; 21.3], *p* = 0.0010) (Fig. 3a). At rest, the adjusted proportion of responders measured by ILA was significantly greater in the aboBoNT-A group (Group A) compared with placebo (Group B) until day 113; no statistical difference between these groups was observed at subsequent time points (*p* = 0.0230) (Fig. 3c).

When comparing assessment methods, there was a statistically significant positive correlation between the ILA and SSA of glabellar lines data (Spearman’s correlation coefficient; *p *< 0.0001 for the aboBoNT-A Group A, placebo Group B, and active comparator Group C [SSA data not reported], and *p* = 0.0258 for placebo Group D [SSA data not reported]).

There was a statistically significant improvement in the appearance of glabellar lines after treatment with aboBoNT-A compared with placebo based on the proportion of responders by the SGA score (85.1% in Group A compared with 1.3% in Group B; *p *< 0.0001) (Table 2), and by the least-squares (LS) mean (standard error [SE]) change in SGA score (2.549 [0.084] compared with −0.053 [0.140], respectively; *p *< 0.0001), which represented a  > 50% improvement from baseline scores based on this model. At all other study visits, the adjusted proportion of responders by SGA score was statistically significantly greater in the aboBoNT-A group (Group A) compared with placebo (Group B) (*p *< 0.0001 at all time points). Overall, there was poor agreement between the IRA of photographs and the ILA to assess the severity of glabellar lines at baseline (35.1%; weighted kappa 0.139 [95% CI 0.103; 0.175]) and day 29 (49.1%; weighted kappa 0.407 [95% CI 0.354; 0.460]). For the adjusted proportion of responders by IRA of photographs at day 29, aboBoNT-A showed statistical superiority over matching placebo in improving the appearance of glabellar lines (Table 2). IRA of photographs at maximum frown at cycle 1, day 85 was statistically significantly greater in the aboBoNT-A group (Group A) compared with placebo (Group B) (93.3% [95% CI 87.4; 96.5] compared with 13.5% [95% CI 5.1; 30.9]; *p *< 0.0001), indicating the superiority of aboBoNT-A over placebo.

**Table 2:** Proportion of responders at cycle 1 day 29 by the IRA of photographs and SGA score of glabellar lines at maximum frown

Assessment	Treatment group^a^			
	Group A	Group B	Group C	Group D
	*N* = 294	*N* = 60	*N* = 94	*N* = 19
* Responder by IRA of photographs*				
Active treatment compared with placebo				
Adjusted proportion of responders, % (95% CI)	99.1 (95.0; 99.8)	25.3 (11.6; 46.7)	94.4 (85.0; 98.1)	31.5 (9.4; 67.1)
Difference (95% CI) aboBoNT-A to matching placebo	–	73.8 (59.1; 88.4)^b^	–	–
Difference (95% CI) active comparator to matching placebo	–	–	–	62.9 (34.9; 91.0)
AboBoNT-A compared with active comparator				
Adjusted proportion of responders, % (95% CI)	97.1 (92.4; 98.9)	–	98.7 (92.9; 99.8)	–
Difference (95% CI) aboBoNT-A to active comparator	–	–	−1.5 (−5.3; 2.2)	–
* Responder by SGA score*				
Active treatment compared with placebo				
Adjusted proportion of responders, % (95% CI)	85.1 (80.2; 88.9)	1.3 (0.2; 9.0)	85.2 (74.4; 91.9)	2.4 (0.2; 23.3)
Difference (95% CI) aboBoNT-A to matching placebo	–	83.7 (78.7; 88.7)^b^	–	–
Difference (95% CI) active comparator to matching placebo	–	–	–	82.8 (72.9; 92.7)
AboBoNT-A compared to active comparator				
Adjusted proportion of responders, % (95% CI)	85.4 (80.5; 89.2)	–	85.4 (76.6; 91.2)	–
Difference (95% CI) aboBoNT-A to active comparator	–	–	−0.0 (−8.2; 8.2)	–

^a^Treatment group in cycle 1. ^b^*p *< 0.0001

Data are shown for the mITT population. Treatment difference and 95% CI s for the treatment comparisons were based on separate multivariate logistic regression models, with treatment group, stratification factors, and center as explanatory variables, and responder (yes or no) as response variable. A responder was defined as having a severity grade of moderate (2) or severe (3) at maximum frown at baseline (day 1), and a severity grade of none (0) or mild (1) at maximum frown at day 29. AboBoNT-A = abobotulinumtoxinA; CI = confidence interval; Group A = patients who received aboBoNT-A 50 U; Group B = patients who received matching aboBoNT-A placebo; Group C = patients who received onaBoNT-A 20 U; Group D = patients who received matching onaBoNT-A placebo; IRA = independent reviewer’s assessment; mITT = modified intent-to-treat; onaBoNT-A = onabotulinumtoxinA; SGA = subject’s global assessment; U = unit

**Fig. 4:**
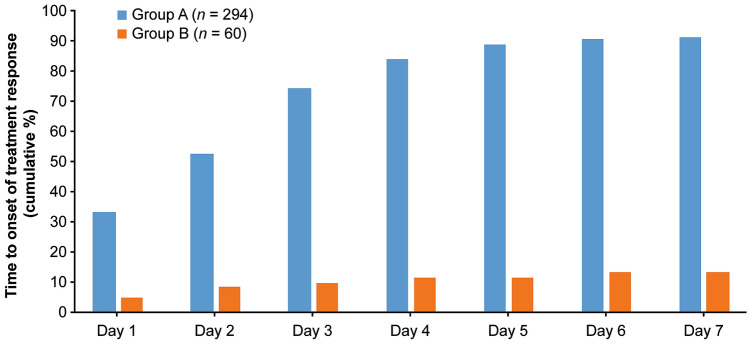
Median time to onset of treatment response according to patients’ diary cards. Data are shown from the mITT population. In Group A, 26 (8.8%) patients reported no response, compared with 52 (86.7%) in Group B by day 7. AboBoNT-A = abobotulinumtoxinA; Group A = patients who received aboBoNT-A 50 U; Group B = patients who received matching aboBoNT-A placebo; mITT = modified intent-to-treat; *n* = number of patients; U = unit

At all visits up to day 85, the LS mean (SE) change in the patient’s self-perception of age from baseline decreased (≤−2.035) in the aboBoNT-A group (Group A) and increased ( ≥ 0.344) in the placebo group (Group B), and there was a statistically significant difference between the groups (*p *< 0.0001 at all time points), indicating that compared with placebo, patients perceived themselves to look younger after aboBoNT-A treatment. Based on patients’ diary card data, the likelihood of responding to treatment was statistically significantly higher for aboBoNT-A (Group A) compared with placebo (Group B) (hazard ratio: 19.33 [95% CI 9.47; 39.46]), and the median time to onset of efficacy was 2.0 days (95% CI 2.0; 3.0) for aboBoNT-A, with 33.3% of patients achieving a response at day 1 (Fig. 4).

### Open-Label Efficacy Results

The median duration between treatment cycles for patients treated with aboBoNT-A was 140.0, 146.0, and 139.0 days for cycles 1, 2, and 3, respectively (i.e., approximately 20 weeks/5 months). The median duration of cycle 4 was 112.0 days, as patients had completed 15 months of treatment (from the start of the cycle 1 period), thus completing the study. Given retreatment depended on glabellar line severity, the number of patients decreased at each cycle, with only one-fifth of patients requiring five treatment cycles within the 15-month study period (Fig. 1). The majority of patients studied here received three treatment cycles.

**Fig. 5:**
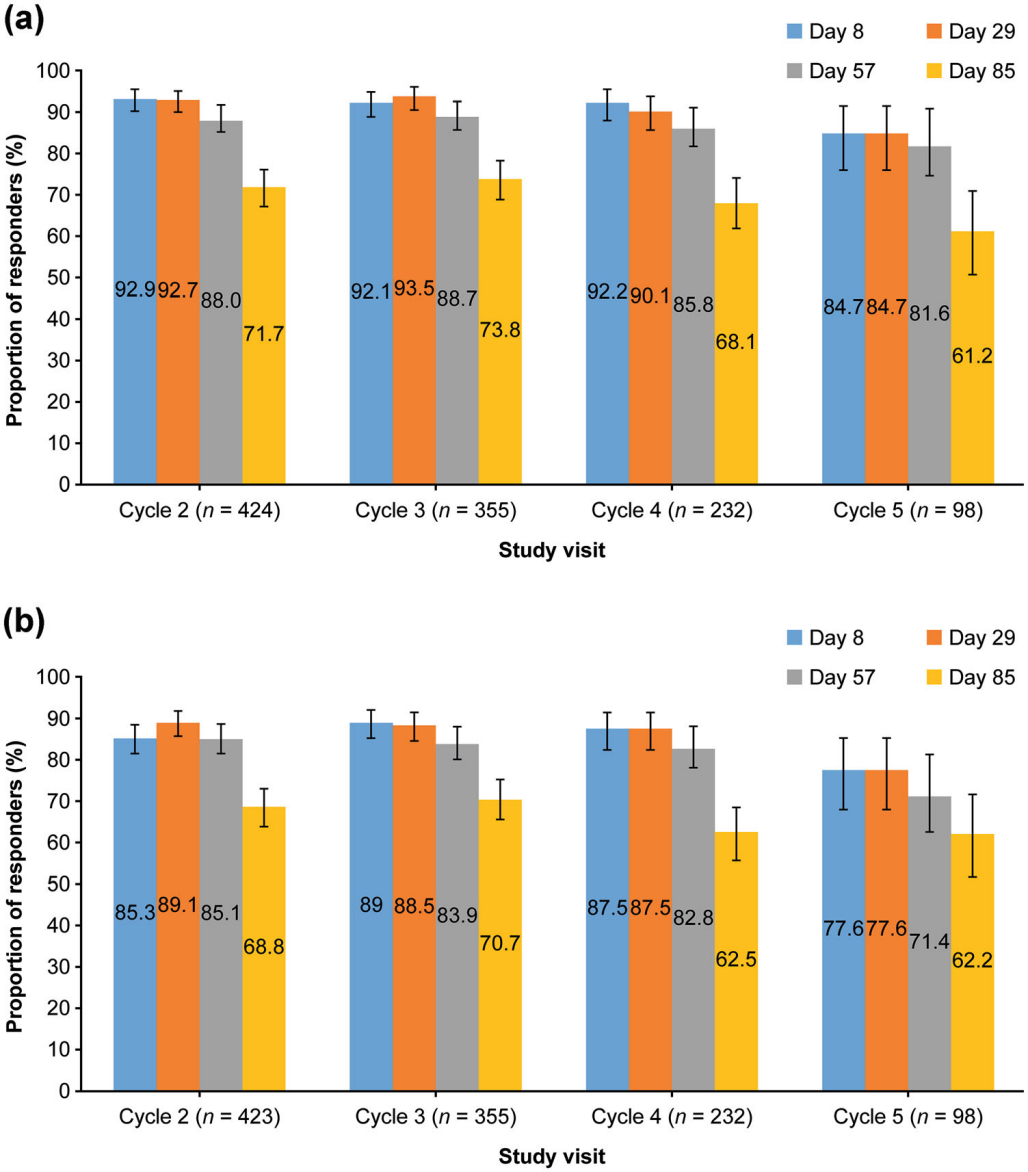
Proportion of responders at days 8, 29, 57, and 85 in cycles 2–5 by **a** ILA of glabellar lines at maximum frown and **b** SSA of glabellar lines at maximum frown. Data are shown from the mITT population. Error bars show 95% CI (calculated using binomial parameter exact method). CI = confidence interval; ILA = investigator’s live assessment; SSA = subject’s self-assessment

At maximum frown, efficacy was maintained over multiple open-label treatment cycles between day 8 and day 29 (when all patients received aboBoNT-A); the proportion of responders was stable across treatment cycles (ILA:  > 90% in cycles 2–4 and 84.7% in cycle 5; SSA:  > 85% in cycles 2–4 and 77.6% in cycle 5) (Fig. 5). At rest, efficacy was also maintained over multiple treatment cycles. In cycles 2 and 3, the proportion of responders by ILA increased slightly from day 8 to day 29 (cycle 2, 60.6% and 63.3%, respectively; cycle 3, 52.2% and 58.0%, respectively), whereas in cycle 4, the proportion of responders was stable from day 8 to day 57 at 54.5%.

The patients’ perception of their age improved (i.e., perceived to look younger) from baseline after multiple cycles of aboBoNT-A treatment: the mean (SD) change from baseline in patients’ self-perception of age at day 29 was −1.9 (3.0) in cycle 2, −1.6 (2.9) in cycle 3, −1.6 (3.0) in cycle 4, and −1.1 (2.4) in cycle 5, albeit with a small number of patients (*n* = 102). Duration of effect was sustained after multiple treatment cycles with aboBoNT-A and was consistent across treatment cycles (approximately 4–5 months), depending upon individual patient response (Table 3).

**Table 3.** Time to retreatment^a^

Treatment cycle duration	Double-blind period	Open-label period	
	Cycle 1 (*N* = 326)	Cycle 2 (*N* = 465)	Cycle 3 (*N* = 385)
Median (95% CI) treatment cycle duration, days^b^	140.0 (134.0; 147.0)	146.0 (140.0; 164.0)	139.0 (133.0; 140.0)

^a^For each treatment cycle, the duration (time to retreatment) was calculated as the time between injection of current cycle and the first injection into next cycle. For a given cycle, if a patient discontinued with no retreatment, this patient was censored at the date of discontinuation for that cycle. ^b^The median and 95% CI are presented using Kaplan–Meier estimate. Data are shown for all randomized patients who received aboBoNT-A 50 U in at least one injection site regardless of the amount administered (safety population). Data for cycle 4 have not been included, as this cycle was shorter, reflecting the fact that these patients had completed 15 months of treatment (from the start of the double-blind period), thus completing the study. The duration of cycle 5 was not included since all patients had completed the study by day 85 of cycle 5.

AboBoNT-A = abobotulinumtoxinA; CI = confidence interval; U = unit

## Safety Results

No new safety signals for aboBoNT-A were reported in this study. The proportion of patients who experienced at least one TEAE during the double-blind cycle was similar between aboBoNT-A and active comparator treatment groups (46.6% and 44.9%, respectively), and the most frequently reported TEAEs were upper respiratory tract infection (URTI; 8.9% and 11.2%, respectively) and viral URTI (4.6% and 3.7%, respectively) (Table 4). The majority of TEAEs reported during the study were non-serious and of mild-to-moderate intensity. Serious TEAEs were reported in <2% of patients and were not considered to be treatment-related. The incidence of treatment-related TEAEs was also similar between aboBoNT-A and active comparator groups (15.6% and 12.1%, respectively), and represented local effects of BoNT-A injections. The most frequently reported treatment-related TEAEs in the aboBoNT-A and the active comparator treatment group, respectively, were eyelid ptosis (4.0% [*n* = 13/326] and 0.9% [*n* = 1/107]), eyelid edema (2.1% [*n* = 7/326] and 0.9% [*n* = 1/107]), injection-site pruritus (1.2% [*n* = 4/326] and 1.9% [*n* = 2/107]), injection-site swelling (1.2% [*n* = 4/326] and 0.0% [*n* = 0/107]), headache (2.8% [*n* = 9/326] and 0.9% [*n* = 1/107]), and injection-site pain (0.9% [*n* = 3/326] and 0.0% [*n* = 0/107]). During the double-blind period, the majority of events of eyelid ptosis were mild in intensity, occurred within approximately 2 weeks of treatment, and resolved within 1 month without any concomitant treatment.

**Table 4:** Treatment-emergent adverse events

TEAEs	Double-blind period				Open-label period			
	Cycle 1				Cycle 2	Cycle 3	Cycle 4	Cycle 5
*n*, (%)	Group A	Group B	Group C	Group D	AboBoNT-A 50 U	AboBoNT-A 50 U	AboBoNT-A 50 U	AboBoNT-A 50 U
	*N* = 326	*N* = 66	*N* = 107	*N* = 21	*N* = 465	*N* = 385	*N* = 244	*N* = 102
Any TEAEs	152 (46.6)	14 (21.2)	48 (44.9)	4 (19.0)	167 (35.9)	129 (33.5)	69 (28.3)	21 (20.6)
Any treatment-related TEAEs^a^	51 (15.6)	0	13 (12.1)	0	32 (6.9)	19 (4.9)	11 (4.5)	1 (1.0)
Intensity of TEAEs								
Mild	112 (34.4)	11 (16.7)	33 (30.8)	4 (19.0)	118 (25.4)	97 (25.2)	53 (21.7)	19 (18.6)
Moderate	35 (10.7)	3 (4.5)	10 (9.3)	0	35 (7.5)	28 (7.3)	13 (5.3)	2 (2.0)
Severe	4 (1.2)	0	3 (2.8)	0	10 (2.2)	1 (0.3)	2 (0.8)	0
Missing	1 (0.3)	0	2 (1.9)	0	4 (0.9)	3 (0.8)	1 (0.4)	0
Any serious TEAEs	5 (1.5)	2 (3.0)	2 (1.9)	0	16 (3.4)	9 (2.3)	5 (2.0)	3 (2.9)
Any TEAEs leading to withdrawal	0	0	2 (1.9)	0	1 (0.2)	1 (0.3)	0	0
Any TEAEs leading to death	0	0	0	0	0	0	0	0
TEAEs reported in ≥ 2% of patients^b,c^								
Eye disorders	32 (9.8)	1 (1.5)	7 (6.5)	0	20 (4.3)	14 (3.6)	5 (2.0)	3 (2.9)
Eyelid edema	7 (2.1)	0	1 (0.9)	0	3 (0.6)	2 (0.5)	0	1 (1.0)
Eyelid ptosis	13 (4.0)	0	1 (0.9)	0	9 (1.9)	6 (1.6)	1 (0.4)	0
Infections and infestations	70 (21.5)	9 (13.6)	24 (22.4)	0	78 (16.8)	60 (15.6)	28 (11.5)	5 (4.9)
URTI	29 (8.9)	4 (6.1)	12 (11.2)	0	34 (7.3)	33 (8.6)	14 (5.7)	3 (2.9)
Viral URTI	15 (4.6)	3 (4.5)	4 (3.7)	0	14 (3.0)	9 (2.3)	3 (1.2)	0
Metabolism and nutrition disorders	7 (2.1)	0	1 (0.9)	0	4 (0.9)	7 (1.8)	3 (1.2)	2 (2.0)
Hyperlipidemia	5 (1.5)	0	1 (0.9)	0	3 (0.6)	6 (1.6)	2 (0.8)	2 (2.0)
Nervous system disorders	16 (4.9)	0	2 (1.9)	0	11 (2.4)	11 (2.9)	6 (2.5)	0
Headache	9 (2.8)	0	1 (0.9)	0	3 (0.6)	3 (0.8)	4 (1.6)	0

Data are shown for all randomized patients who received aboBoNT-A 50 U in at least one injection site regardless of the amount administered (safety population). ^a^Relationship to study drug was assessed by the Investigator. ^b^TEAEs with preferred term reported in  ≥ 2% of patients treated with aboBoNT-A 50 U in any treatment cycle. ^c^If a patient experienced more than one event in a category, the patient is counted only once in that category. AboBoNT-A = abobotulinumtoxinA; Group A = patients who received aboBoNT-A 50 U; Group B = patients who received matching aboBoNT-A placebo; Group C = patients who received onaBoNT-A 20 U; Group D = patients who received matching onaBoNT-A placebo; *N* = number of total patients; *n* = number of patients with events; onaBoNT-A = onabotulinumtoxinA; SOC = system organ class; TEAE = treatment-emergent adverse event; U = unit; URTI = upper respiratory tract infection

In the open-label period, there was a trend toward a decreased incidence of TEAEs and treatment-related TEAEs with repeated cycles of aboBoNT-A 50 U received over a total treatment duration of 15 months. The incidence of treatment-related eyelid ptosis (1.9%, 1.6%, 0.4%, and 0% of patients in cycles 2, 3, 4, and 5, respectively) and eyelid edema (0.6%, 0.5%, 0.0%, and 1.0% of patients in cycles 2, 3, 4, and 5, respectively) decreased overall with repeated treatment of aboBoNT-A.

During cycle 1, two patients (1.9%) withdrew from the active comparator group (one case of mild eyelid ptosis [non-serious, treatment-related TEAE] and one case of mild eczema [non-serious, unrelated TEAE]). During the open-label phase, two patients (0.4%) treated with aboBoNT-A 50 U withdrew as a result of TEAEs, both unrelated to study treatment (one case of invasive breast carcinoma [serious; cycle 2], and one case of optic neuritis [non-serious; cycle 3]).

No TEAEs suggestive of remote spread of toxin effects were identified during the study. One patient experienced swelling of the face suggestive of a hypersensitivity reaction in cycle 1, which may have been confounded by concomitant urticaria chronica. The majority of injection-site reactions occurred during cycle 1, the most common being injection-site pruritus (four patients [1.2%] in the aboBoNT-A group and two patients [1.9%] in the active comparator group), with the incidence decreasing after subsequent treatment cycles with aboBoNT-A. The majority of injection-site reactions occurred on the day of injection or the day following injection and resolved quickly within 1–3 weeks.

No patient showed a seroconversion for binding antibodies or neutralizing antibodies to BoNT-A during the study. Both binding and neutralizing antibodies were detected at baseline in one patient who received aboBoNT-A 50 U; the patient tested negative at cycle 1, day 85, and then positive again at end of study for binding and neutralizing antibodies. The latter finding was unexpected but was not considered to be related to administration of the active product.

## Discussion

This phase 3, randomized, double-blind study demonstrated that a single injection of aboBoNT-A 50 U was superior to matching placebo in improving the appearance of glabellar lines at maximum frown at day 29 (95% compared with 1%; per ILA), and non-inferior to active comparator (onaBoNT-A 20 U; per ILA) in Chinese adults. Efficacy and responder rates in this population were similar to those in a study conducted in 2010 assessing botulinum toxin to treat glabellar lines in Chinese patients [22]. These results also support previous studies in which aboBoNT-A injections consistently reduced the severity of glabellar lines [7, 12, 18, 23] and demonstrate the efficacy of aboBoNT-A in a new patient population, thus adding to the existing body of evidence in favor of aboBoNT-A.

The proportion of patients who had severe glabellar lines at baseline (56.9%) in the current study was similar with previous studies investigating aboBoNT-A 50 U in glabellar lines enrolling mainly Caucasian patients: 48.6%, 62.4%, and 56.2% in studies by Ascher et al. (2018), Monheit et al. (2007), and Rzany et al. (2006; five injection sites treatment arm), respectively [15, 23, 24]. Despite the similarity in glabellar line severity in these patient populations, responder rates after aboBoNT-A treatment appear higher in the current study (ILA assessment: 95%, IRA assessment: 99%, SGA score at day 29: 85% [at least a 50% improvement in assessment of glabellar lines]) than previous studies of largely Caucasian populations. Responder rates (by ILA, unless indicated) after aboBoNT-A treatment at day 30 were 89.0% in the Monheit et al. (2020) study, 83.7% in Moy et al. (2009), 89.5% in Brandt et al. (2009), 83.7% in Carruthers et al. (2002), 7.1% at day 29 in Ascher et al. (2018), and 86.3% at week 4 in the Rzany et al. (2006, IRA) study [6, 7, 12, 15, 18, 24]. The higher responder rates in the current study could be due to differences in facial anatomy; for example, Chinese and Korean people possess different glabellar contraction patterns compared with Caucasian people [25]. In addition, the mass of some muscles, such as the corrugators, is often lower in Asian patients than in Caucasian patients, and the corrugators tend to be shorter, narrower, and less hyperdynamic in Asian people compared with Caucasian people, resulting in less severe glabellar lines [2, 25].

In the current study, patients also reported statistically significant improvements in the perception of their age (i.e., they perceived that they looked younger) after treatment with aboBoNT-A compared with placebo during cycle 1, and after multiple cycles of treatment. This is supported by Chang et al. (2016), whereby patients reported that they believed they looked an average of 5.6 years younger after aboBoNT-A treatment [26], and by the APPEAL study, in which the percentage of patients who perceived themselves to look younger ranged from 82.9% after one injection to 91.9% after three injections with aboBoNT-A [27]. Similarly, Chinese patients treated with onaBoNT-A reported that they felt their treatment made them look an average of 2.1 years younger than their chronological age after day 90 of treatment [22].

Patients and investigator assessments were concordant in this study. Patients reported that response to treatment occurred after a median of 2.0 days post-aboBoNT-A treatment during cycle 1, with 33% of patients responding to treatment at day 1. These results are supported by other studies showing rapid onset of response, with studies by Moy et al. (2009) and Brandt et al. (2009) reporting the median time to onset of effect as early as 3 days after treatment with aboBoNT-A, and Monheit et al. (2020) at day 2 [7, 12, 18]. Karbassi et al. (2018) also reported that 87.5% of patients responded to treatment within the first 48 hours after aboBoNT-A injection [28], and Kassir et al. (2013) reported that 28% of patients treated with aboBoNT-A reported improvement within 1 day [17]. The efficacy of aboBoNT-A was maintained across multiple open-label treatment cycles and was generally similar across cycles 2–4. This is consistent with the study by Moy et al. (2009), in which the investigator-assessed response rate (maximum frown) ranged from 80% to 91% during up to 5 cycles of aboBoNT-A 50 U in an open-label assessment of 1200 patients [7, 29]. Duration of effect was sustained after multiple treatment cycles with aboBoNT-A 50 U and was consistent across cycles, varying between approximately 4 months and 5 months, which is in line with previous research and translates to only three injections required per year to maintain treatment effect [7, 16, 17, 30]. Longer time to retreatment provides a significant advantage to patients with regard to reducing the number of injections, leading to reduced burden and costs.

The agreement between responder rates measured by ILA and IRA of photographs was generally limited, which may suggest that photographs are less suitable than live assessment to assess the effect of aboBoNT-A on the appearance of dynamic glabellar lines. The disparity between the proportion of male and female participants enrolled in the study may also be considered a limitation; however, this is a known difficulty in aesthetic studies. The results from cycle 5 must also be interpreted with caution, as the study design meant that a relatively small number of patients required five treatment cycles within the planned follow-up period of 15 months.

Single and repeated injections with aboBoNT-A administered over a maximum of 15 months were well-tolerated and similar to that seen in other patient populations and ethnicities [7, 13, 14, 24]. The safety profile observed was as expected based on the pharmacology of the compound and the treated indication, and no new safety concerns were identified. The most frequently reported treatment-related TEAEs in the eye were eyelid ptosis and eyelid edema, which are expected TEAEs consistent with the mechanism of action of botulinum toxins. The incidence of eyelid ptosis and edema generally declined across treatment cycles, which may have been due to physicians improving their injection technique. Safety in this population was also similar to that seen in previous studies of Chinese patients [22].

## Conclusions

In conclusion, single and multiple treatments with aboBoNT-A 50 U were shown to be clinically more efficacious compared with placebo in improving the appearance of moderate to severe glabellar lines in Chinese patients. In addition, responder rates with aboBoNT-A (assessed by both the investigator and patient) were non-inferior to an active comparator at day 29 after a single injection. The results demonstrate that aboBoNT-A was well-tolerated with no new safety signals and had a rapid onset of efficacy and a long duration of effect, which were repeatable over treatment cycles.

Given that limited clinical data are available on the safety and efficacy of aboBoNT-A for the treatment of moderate-to-severe glabellar lines in the Chinese population, the results from this study help to address this gap, providing clinical experience and evidence regarding the use of aboBoNT-A in China.

## Ethical Approval

The study was conducted under the provisions of the Declaration of Helsinki, independent ethics committees, China’s Ethics Committee Guidelines, informed consent regulations, and in accordance with the International Conference on Harmonisation, Good Clinical Practice (GCP), China GCP Guidelines, and adhered to all applicable local regulatory requirements. All patients gave written informed consent prior to study enrollment. All procedures performed in studies involving human participants were in accordance with the ethical standards of the institutional and/or national research committee and with the 1964 Helsinki declaration and its later amendments or similar ethical standards.

## Data Availability

Anonymized patient-level trial data that underlie the results reported in this publication may be made available to researchers who provide a research proposal. Data from eligible studies are available 6 months after the studied medicine and indication have been approved in the USA and EU or after the primary manuscript describing the results has been accepted for publication, whichever is later. Additional study documentation including the clinical study report, study protocol with any amendments, annotated case report form, statistical analysis plan, and dataset specifications may also be made available. Patient-level data will be anonymized, and study documents will be redacted to protect the privacy of trial participants. Any requests should be submitted to www.vivli.org for assessment by an independent scientific review board. Further details on Ipsen’s sharing criteria, eligible studies, and process for sharing are available here (https://vivli.org/members/ourmembers/).
