# Correction: Efficacy and Safety of AbobotulinumtoxinA for the Treatment of Glabellar Lines in Chinese Patients: A Pivotal, Phase 3, Randomized, Double-Blind and Open-Label Phase Study

**DOI:** 10.1007/s00266-023-03306-1

**Published:** 2023-03-23

**Authors:** Yan Wu, Fang Fang, Wei Lai, Chengxin Li, Li Li, Quanzhong Liu, Jianyun Lu, Xiaowen Pang, Jiaming Sun, Xiaofeng Shi, Philippe Picaut, Inna Prygova, Bill Andriopoulos, Qiuning Sun

**Affiliations:** 1 Peking University First Hospital, No. 8 Xishiku Street, Xicheng, Beijing, 100034 China; 2 Institute of Dermatology, Chinese Academy of Medical Sciences, Nanjing, China; 3 Department of Dermatology, The Third Affiliated Hospital of Sun Yat-Sen University, Guangzhou, China; 4 General Hospital of People’s Liberation Army (301 Hospital), Beijing, China; 5 West China Hospital, Sichuan University, Chengdu, China; 6 Tianjin Medical University General Hospital, Tianjin, China; 7 Third Xiangya Hospital of Central South University, Changsha, China; 8 PLA Air Force General Hospital, Beijing, China; 9 Union Hospital, Tongji Medical College, Huazhong University of Science and Technology, Wuhan, China; 10 Ipsen, Shanghai, China; 11 Ipsen, Cambridge, MA USA; 12 Ipsen, Boulogne-Billancourt, France; 13 Galderma, Uppsala, Sweden; 14 Peking Union Medical College Hospital, Beijing, China

**Correction to: Aesth Plast Surg (2023) 47:351–364** https://doi.org/10.1007/s00266-022-03164-3

This article was updated to replace Fig. 2.

